# Supplementary material for: Synthesis and Characterization of Lithium-Ion Conductive LATP-LaPO4 Composites Using La2O3 Nano-Powder
Source: Materials (Basel). 2021 Jun 23;14(13):3502. doi: 10.3390/ma14133502 (PMC8269504; doi:10.3390/ma14133502)
Supplement: Supplementary file 1 [file materials-14-03502-s001.zip › Supplementary Materials4.pdf]

Supplementary Materials for Synthesis and Characterization of Lithium-Ion Conductive LATP-$LaPO_4$ Composites Using La_2O_3 Nano-Powder

Fangzhou SONG[1], Masayoshi UEMATSU[1], Takeshi YABUTSUKA[1], Takeshi YAO[2], Shigeomi TAKAI[1,*]

[1]Graduate School of Energy Science, Kyoto University, Yoshida-Honmachi, Sakyo-Ku, Kyoto, Japan

[2]Kyoto University, Yoshida-Honmachi, Sakyo-Ku, Kyoto, Japan

To optimize the sintering time for LATP – La_2O_3 composites, a LATP – 8wt.% La_2O_3 was sintered with different durations from 1 to 10 hours. Fig. S1 shows the powder XRD pattern of LATP – 8wt.% La_2O_3 with different sintering times, where the impurity is generally unchanged despite longer sintering durations. In addition, sintering longer than 4 hours can promote particle growth which leads to the severe particle aggregation as shown in Fig. S2. The conductivity of LATP – 8wt.% La_2O_3 is plotted as a function of sintering time in Fig. S3, which is decreased with the sintering time. Therefore, in consideration of overall impurity level, particle growth and sample conductivity, a four-hour sintering time was selected for the sample preparations in this work.

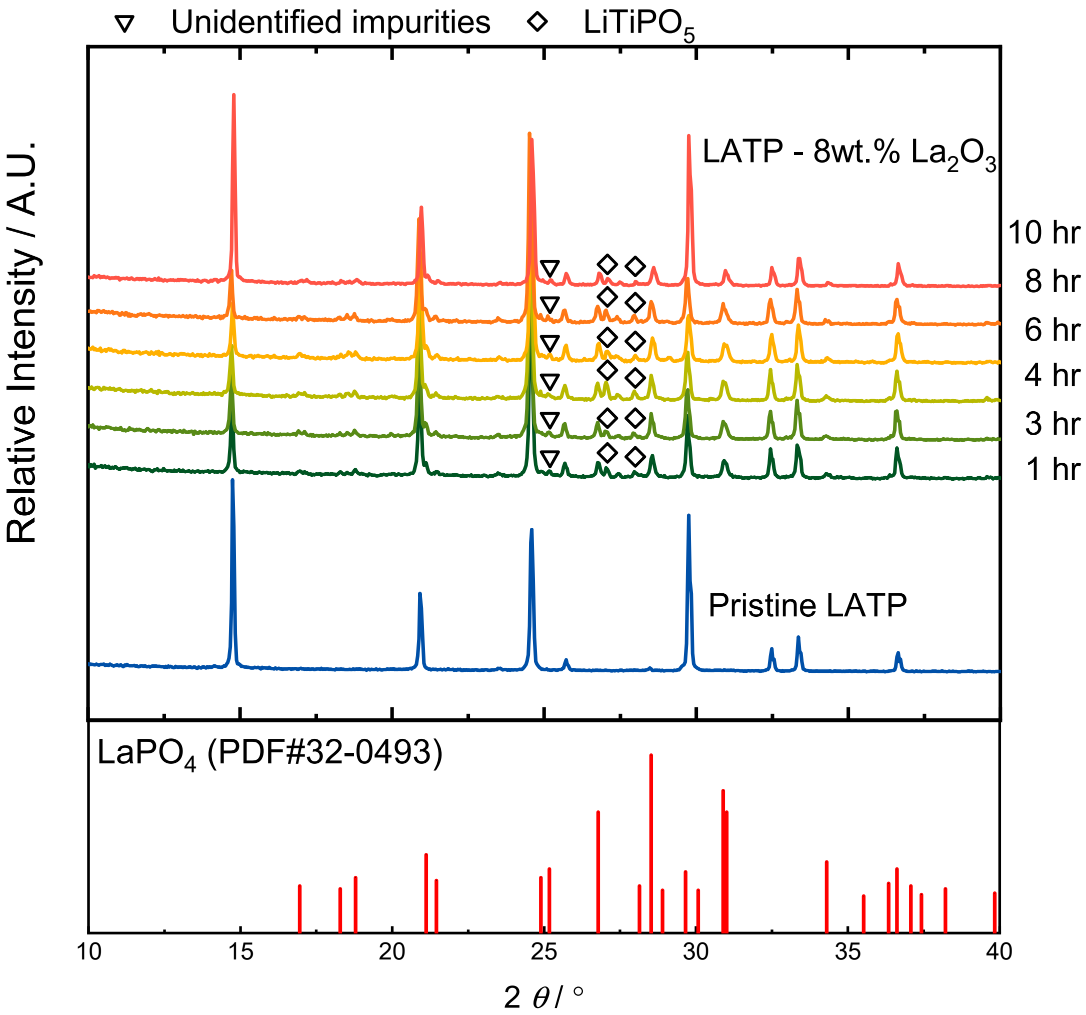

Figure S1 Powder XRD pattern of LATP – 8wt% La_2O_3 sample with sintering times ranging from 1 to 10 hours, the unidentified impurity and $LiTiPO_5$ peaks are labeled by hollow inverted triangles and hollow diamonds.

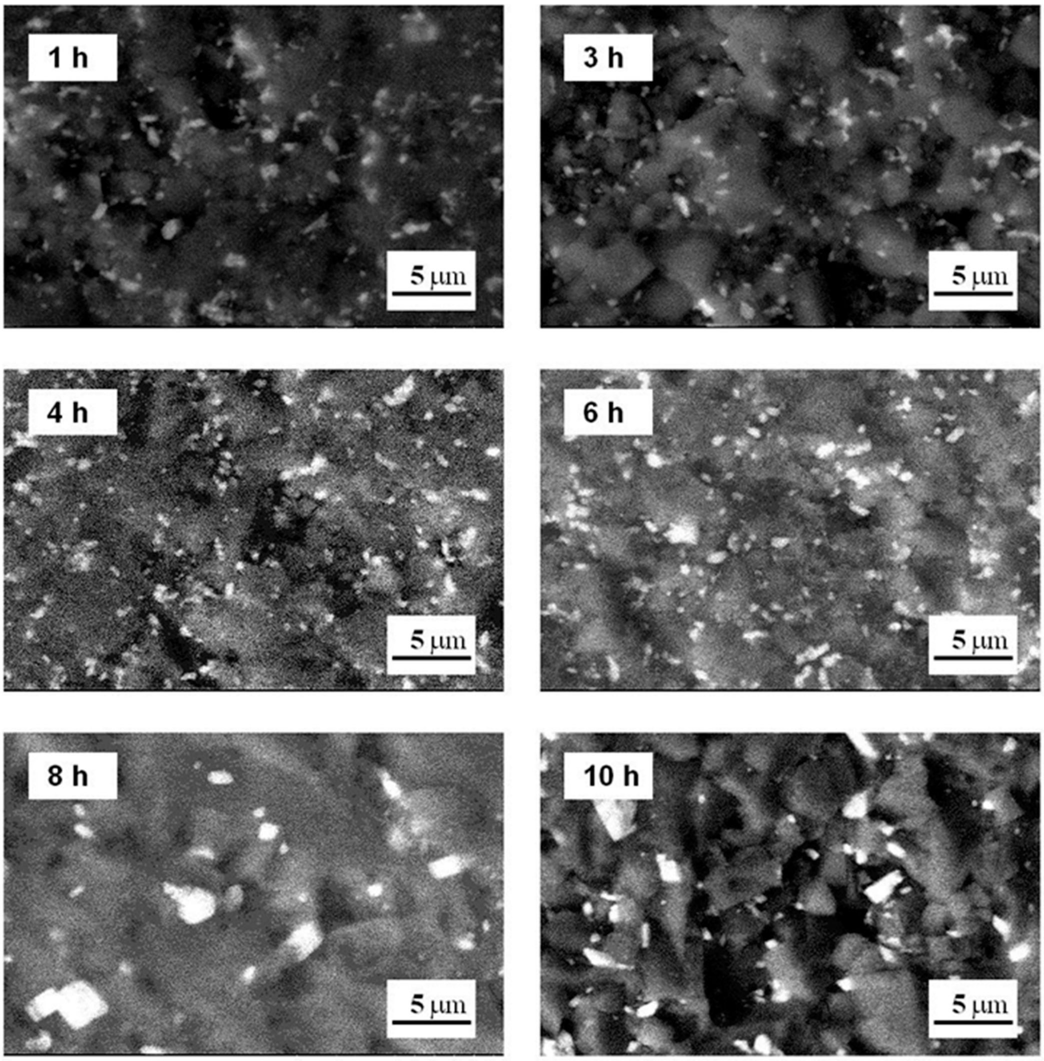

Figure S2 Back-scattered SEM images of LATP – 8wt.% La_2O_3 samples with different sintering times.

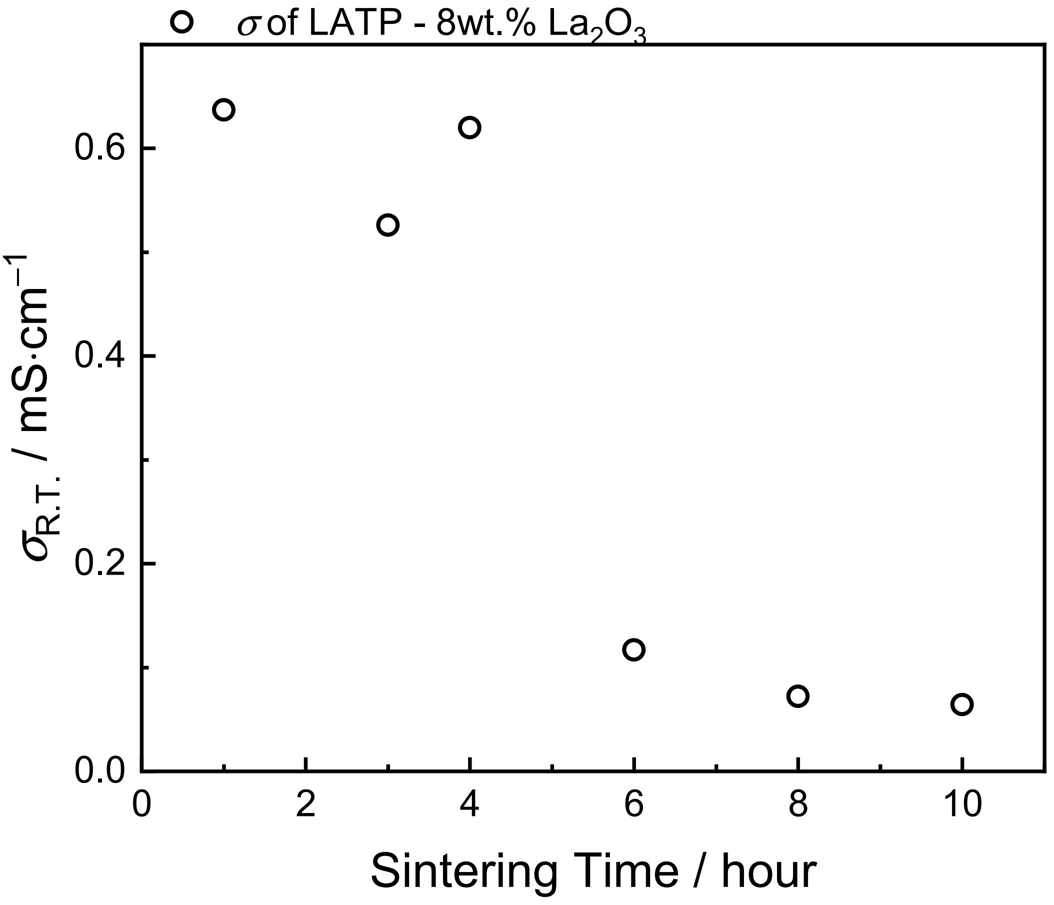

Figure S3 Conductivity of LATP – 8wt.% La_2O_3 as a function of sintering time.
